# Effect of Third and Fourth mRNA-Based Booster Vaccinations on SARS-CoV-2 Neutralizing Antibody Titer Formation, Risk Factors for Non-Response, and Outcome after SARS-CoV-2 Omicron Breakthrough Infections in Patients on Chronic Hemodialysis: A Prospective Multicenter Cohort Study

**DOI:** 10.3390/jcm11113187

**Published:** 2022-06-02

**Authors:** Frank-Peter Tillmann, Lars Figiel, Johannes Ricken, Hermann Still, Christoph Korte, Grete Plaßmann, Ana Harth, Achim Jörres, Philipp von Landenberg

**Affiliations:** 1 Department of Medicine I—Nephrology, Transplantation & Medical Intensive Care, Medical Center Cologne-Merheim, University Witten/Herdecke, Ostmerheimer Str. 200, D-51109 Cologne, Germany; hartha@kliniken-koeln.de (A.H.); joerresa@kliniken-koeln.de (A.J.); 2 Nephrologisches Zentrum Ibbenbüren, Gravenhorsterstr. 1, D-49477 Ibbenbüren, Germany; still@dialysen-muensterland.de; 3 Nephrologisches Zentrum Emsdetten, Nordwalderstr. 48-50, D-48282 Emsdetten, Germany; figiel@dialysen-muensterland.de (L.F.); korte@dialysen-muensterland.de (C.K.); 4 Nephrologisches Zentrum Rheine, Neuenkirchenerstr. 104, D-48431 Rheine, Germany; ricken@dialysen-muensterland.de (J.R.); plassmann@dialysen-muensterland.de (G.P.); 5 LADR GmbH MVZ Nord-West, Technikerstr. 14, D-48465 Schüttorf, Germany; p.landenberg@ladr.de

**Keywords:** SARS-CoV-2, COVID-19, omicron, vaccination failure, neutralizing antibodies, hemodialysis, booster vaccination, breakthrough infection

## Abstract

The aim of this study is to determine the effect of repeated vaccinations on neutralizing SARS-CoV-2 IgG antibody titers, evaluate risk factors for immunological non-response, and to report breakthrough infections in chronic hemodialysis patients. Methods: A prospective, multi-center cohort study in 163 chronic hemodialysis patients was conducted. Antibody titers were measured three months after second, third, and fourth (10 pts) booster vaccinations. SARS-CoV-2 neutralizing antibody titers in BAU/mL and % inhibition were divided into three categories (<216, 216–433, >433 and <33, 33–66, and >66%). Somers’s test, paired t-test, and univariable and multivariable logistic regression analysis were applied to evaluate differences in antibody levels and search for risk factors for vaccination failure defined as neutralizing titers <50% and/or need for repeated booster vaccinations. Furthermore, we report on a case series to describe characteristics of patients after four vaccinations (*n* = 10) and breakthrough infections (*n* = 20). Results: Third dose boosters resulted in higher proportions of patients with neutralizing antibody levels >66% as compared to after the second dose (64.7% after second dose vs. 88.9% after third dose, *p* = 0.003), as well as in a respective increase in neutralizing titer levels in % from 68 ± 33% to 89 ± 24 (*p* < 0.001). The proportion of patients with IgG-titers below 216 BAU/mL decreased from 38.6 to 10.5% (*p* ≤ 0.001). Age (*p* = 0.004, OR 1.066, 95% CI 1.020–1.114) and presence of immunosuppressive medications (*p* = 0.002, OR 8.267, 95% CI 2.206–30.975) were identified as major risk factors for vaccination failure. Repeated booster vaccinations ≥4 times were effective in 8 out of 10 former low-responders (80%) without any side effects or safety concerns. Breakthrough infections showed a clinically mild course but were associated with prolonged viral shedding on PCR-testing ranging 7–29 (mean 13) days. Conclusions: Third and fourth mRNA-based booster vaccinations resulted in higher and longer lasting SARS-CoV-2 antibody levels as compared to after two dosages. The presence of immunosuppressive medication and repeat vaccinations are major potentially modifiable measures to increase antibody levels in non-or low-responders. Breakthrough infections with SARS-CoV-2 Omicron were associated with prolonged viral shedding but clinically mild disease courses.

## 1. Introduction

Numerous studies worldwide have so far demonstrated a high physical and psychological burden on dialysis patients during the COVID-19 pandemic, which is now lasting over two years [1]. International investigations performed early during the pandemic reported high case-fatality rates from 20 to 30% [2,3]. Correspondingly high mortality rates have also been published in Germany [4]. Epidemiologic population-based reports have demonstrated a four-fold increase in mortality compared to patients without end-stage renal disease even after adjustment for various cofactors [5,6]. However, high rates of asymptomatic infections in dialysis patients have also been demonstrated in previous studies [7,8]. In the early phase of the pandemic, a short time interval between infection and fatal outcome indicated either medically inadequate initial control of viral load or high comorbidity of affected dialysis patients [9,10]. In the meantime, different virus variants with a potentially different course of infection have been identified worldwide and classified as variants of concern (VOC) [11]. Besides the potential of these variants for an altered clinical course, the probability of an antigen variation induced reduction in vaccine-induced immunity has been a major concern. For example, the currently dominant viral variant B.1.1.52 (omicron) contains over >30 genetic alterations in the spike protein that have been associated with consecutively increased infectivity and ability to escape the immune system [12,13,14]. Recent data from the Robert Koch Institute in Berlin indicate that the omicron variant was the dominant SARS-CoV-2 variant in Germany in March 2022. The proportion of all other variants, including delta, is less than 1% [15]. Therefore, several unanswered questions remain for nephrologist: Will omicron lead to higher infection rates among dialysis patients? How to deal with insufficient immune response after the third vaccine? How will the clinical disease course of after omicron infection be like in hemodialysis patients? For these reasons, data on current IgG antibody titers, titer increases after booster vaccinations, antibody ability to also neutralize the omicron variant, as well as clinical courses after breakthrough infections in dialysis patients are currently of relevance. Therefore, the objectives of this study were (**A**) to assess the effect of third dose booster vaccinations on SARS-CoV-2 IgG antibodies and their respective neutralizing capacity, and (**B**) to search for risk factors for immunological nonresponse in this particularly vulnerable patient population on chronic hemodialysis. Another aim of this investigation was (**C**) to evaluate the effects of multiple ≥4 vaccinations in prior non- or low-responders. In addition (**D**), we report on the clinical course after breakthrough infections with SARS-CoV-2 in a case series of 20 hemodialysis patients.

## 2. Methods

This is a prospective, longitudinal multi-center cohort investigation in chronic hemodialysis patients >18 years of age and part of a project to prospectively evaluate diverse outcome parameters in chronic hemodialysis patients during the SARS-CoV-2 pandemic. Patients were recruited in three hemodialysis centers in Germany. Patients were classified to participate in this study if they were vaccinated with two or more dosages of either mRNA SARS-CoV-2 vaccine (BNT162b2, Pfizer-BioNTech, Mainz, Germany) or replication-defective viral vector carrying pathogen gene (ChAdOx1 nCoV-19, Oxford-AstraZeneca, Oxford, UK). Anti-SARS-CoV-2 antibodies were evaluated several times during the study after the second and third booster vaccination as well as after repeat vaccinations in selected individuals. Post-vaccination analysis included measurement of SARS-CoV-2 IgG-antibody titers and evaluation of the neutralizing capacity of the IgG-antibody as described below. Patients had been vaccinated either in central vaccination facilities, in primary care, or in the dialysis facility itself. Dates of vaccination, type of vaccination used, and person-related data were stored centrally in a password protected data sheet. Patients classified as non- or low-responders were offered a third or even repeated booster vaccination with BNT162b2 after detailed information and written consent. Past medical history of COVID-19 and outcomes before the start of the study were determined by the medical staff of the facility in all participants prior to the start of the study. Demographic data (age, sex, BMI, dialysis vintage, prior history of transplantation, estimated glomerular filtration rate by CKD-EPI formula in ml/min/1.73 m^2^ BSA, albumin in mg/L, time from vaccination to laboratory measurement of SARS-CoV-2 antibody titers, type of hemodialysis access [fistula, graft, catheter], type of dialysis membrane, candidacy for renal transplantation, active immunosuppressive medication at the time of antibody titer evaluation, diabetes mellitus, and active malignancy) were recorded in every patient at baseline. Initially, 233 patients gave informed consent to participate in this investigation. Over the time, 70 patients did not fulfill the criteria to enter the current investigation reported herein: 28 patients had received their first SARS-CoV-2 vaccination prior to entering our chronic hemodialysis program, 11 patients had not received a third booster vaccination, 8 patients were infected with SARS-CoV-2 before the booster vaccination, 2 patients moved, 6 patients denied a booster cycle, 7 died in the meantime, 7 patients were in hospital at the time of laboratory evaluation of SARS-CoV-2 antibodies, and 1 received I kidney transplant. Therefore, the final cohort consisted of 163 patients and a case series of 20 patients with breakthrough infections. The study was approved by the local ethics committee “*Ethikkommission der Ärztekammer Westfalen-Lippe und der Westfälischen Wilhelms Universität*” in Münster (2021-131-f-S) and conducted in line with the Declaration of Helsinki and the European Union Clinical Trials Directive 2001/20/EC (EU CTD). Written informed consent to participate and to publish was obtained from all individual participants included in the study. All patients gave informed consent prior to study participation and before a third booster-vaccination.

### 2.1. Statistical Outcome Parameter, Cohort, and Case Series Definitions

We statistically analyzed two patient groups with respect to non-response after vaccination. The first group (A) consisted of 153 patients without need for repeat vaccination who received standard routine booster vaccinations after approval of the vaccines for booster vaccination in Germany (outcome defined per protocol as “neutralizing antibody titers <50%”). This definition was predefined in the statistical analysis plan before data curation and was used due to lack of standardized antibody titer reporting and large inter-kit variability. Furthermore, neutralizing antibody titers theoretically better reflect vaccination induced protection than IgG antibody titer levels alone. This group was analyzed with respect to the effect of a third booster vaccination on increases in neutralizing antibody titers as compared to antibody generation after the second dose. The second group (B) consisted of 163 participants who had received either a routine third boost vaccination after prior two standard vaccination dosages (153 patients of group (A) and another 10 patients who had not been able to mount sufficient antibody titers and had agreed to repeat vaccinations ≥4 (outcome defined as a composite of “neutralizing antibody titers <50% and/or need for repeat vaccination”). This group was analyzed with respect to risk factors for vaccination failure. Furthermore, we describe two case series. One comprises 10 patients with repeat ≥4 vaccinations (incorporated in the analysis of group (B) and a second describes 20 patients after SARS-CoV-2 breakthrough infections. 

### 2.2. SARS-CoV-2 Antibody Test Assays

*SARS-CoV-2 IgG Antibody Test Assay:* A commercially available immunoassay was used for antibody detection, the anti-SARS-CoV-2 S-RBD IgG (Snibe Diagnostics, New Industries Biomedical Engineering Co., Ltd. [Snibe], Shenzhen, China). SARS-CoV-2 S-RBD IgG is a chemiluminescent immunoassay (CLIA) that determines IgG Ab against the RBD of the Spike (S) protein of the virus, in human serum or plasma. All analyses were performed on MAGLUMI™ 4000 instrument (Snibe Diagnostics), with results expressed in BAU/mL. The assay has a clinical sensitivity between 74.5% (days post onset of Symptoms 0–7) and 100.0% (days post onset of Symptoms >15), and a specificity of 99.6% (95% confidence interval [95% CI] 98.7–100.0%). Results were reported in BAU/mL from 0 to a cut-off level of 433. Values greater than 433 were reported >433 BAU/mL. For analysis, these data were categorized into three classes of IgG-levels of 0–216, 216–433, and >433 BAU/mL. *SARS-CoV-2 IgG* *neutralizing test assay:* We used the ELISA-based GenScript SARS-CoV-2 Surrogate Virus Neutralization Test Kit (GenScript 105 Biotech, Piscataway Township, NJ, USA). The test was used according to the manufacturer’s recommendations. Samples were diluted in sample buffer and incubated at 37° for 30 min in the 96-well microtiter plates provided, followed by the respective wash and incubation cycles, including controls and required reagents. The microtiter plates are coated with the “host cell receptor” angiotensin-converting enzyme 2 (ACE2). Samples containing SARS-CoV-2 neutralizing antibodies block the protein-protein reaction between ACE2 and the added (S)-RBD-horseradish peroxidase conjugate. The reduced color change upon addition of chromogenic substrate can be measured photometrically. Optical density (OD) was measured at 450 nm using the microplate reader of a VIRCLIA^®^ automation system (Vircell, Granada, Spain). The signal to cut-off ratio was calculated and the values printed and interpreted according to the manufacturer’s protocol. Results were reported in %. 

### 2.3. SARS-CoV-2 PCR-Test Assay

RNA was extracted on an MGI SP-960 instrument, a high-throughput fully automated workstation, using the MGIEasy Nucleic Acid Extraction kit (MGI™) and amplified on the QuantStudio™ 5 Real-Time PCR System (Applied Biosystems, Waltham, MA, USA) using the Thermo Fisher^®^ TaqPath™ COVID-19 CE-IVD RT-PCR kit. The TaqPath assay targets three sequences in the virus ORF1ab, N and S genes. The internal control for nucleic acid extraction was an MS2 phage. Results were interpreted using the COVID-19 Interpretive Software Version v.2.5 on QuantStudio™ Design (Applied Biosystems, Waltham, MA, USA) and Analysis Desktop Software Version 1.5.1 (Applied Biosystems, Waltham, MA, USA). Positive results were classified according to cycle threshold (Ct) data obtained for all three targets as described elsewhere. TaqPath™ COVID-19 CE-IVD RT-PCR Kit contains the reagents and controls for a real-time reverse transcription polymerase chain reaction (RT-PCR) test intended for the qualitative detection of nucleic acid from SARS-CoV-2 in upper respiratory specimens (such as nasopharyngeal, oropharyngeal, nasal and mid-turbinate swabs, and nasopharyngeal aspirate) and bronchoalveolar lavage (BAL) specimens from individuals suspected of COVID-19. Positive results are indicative of the presence of SARS-CoV-2 RNA; Negative results do not preclude SARS-CoV-2 infection and should not be used as the sole basis for patient management decisions. Limit of Detection studies for this kit were performed by extracting 400 μL of each specimen followed by elution in 50 μL, then 5 μL of eluate was added to the RT-PCR reaction. The LoD of 10 GCE/reaction is calculated from a starting concentration of 250 GCE/mL of specimen.

### 2.4. Statistical Analyses

Data are shown as mean plus minus standard deviation (SD) or percentage, according to the type of variable analysed. We used the Somers’s test for associations between qualitative ordinal variables and a paired *t*-test for paired quantitative variables. Univariable logistic regression was applied to search for risk factors for vaccination non-response. Variables with assumed impact on the outcome parameters were then further analyzed on multivariable logistic regression analysis (enter-method). Results were cross-checked by sensitivity analyses using a stepwise forward logistic regression model. Values of *p* < 0.05 were considered statistically significant. Statistical analyses were performed using SPSS, IBM Corp., Armonk, NY, USA.

## 3. Results

### 3.1. Effect of a Third Booster Vaccination on SARS-CoV-2 IgG Antibodies and Their Neutralizing Capacity (**A**) 

**Figure 1:**
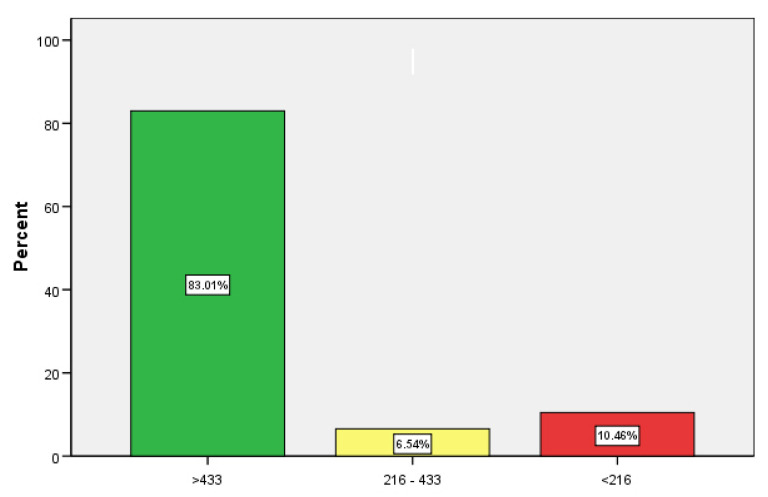
% of participants with SARS-CoV-2 antibodies in categories (BAU/mL) after a third (booster) vaccination in 153 chronic hemodialysis patients.

We investigated the effect of a third booster vaccination in 153 (93.9% of the total cohort) chronic dialysis patients (60.8% males, BMI 26.7 ± 5.5), who had received three dosages of SARS-CoV-2 vaccination. Mean age and mean dialysis vintage were 67.4 ± 15.8 years (min 19.1, max 97.5) and 5.5 ± 5.1 years (min 0.8, max 30.1), respectively. Many patients had been vaccinated in different locations (vaccination centers, primary care practices, or dialysis facilities) and time elapsed between the first and second and between the second and third vaccinations were 41 ± 14 and 166 ± 40 days, respectively. SARS-CoV-2 IgG-antibodies were determined on average 93 ± 63 days after the second and 89 ± 32 days after the third booster vaccination (*p* = 0.415). Nine patients were dialyzed with PMMA membranes, whereas all others were treated with polysulfone dialyzers (94.1%). A total of 12 patients were on hemodialysis after renal transplant failure (7.8%), and 46 were waitlisted (30.1%). Immunosuppressive (CNIs, steroids or chemotherapy) therapy was present in 16 persons (10.5%), 47 were treated for diabetes mellitus (30.7%), 6 suffered from active malignancy (3.9%), and 103 had a native fistula (67.3%). Mean Kt/V was 1.47 ± 0.39, mean estimated glomerular filtration rate was 8 ± 4 in ml/min/m^2^ BSA, and mean albumin was 3676 ± 433 mg/L. Seven patients had received heterologous vaccination schemes with vector and mRNA-based vaccines (4.6%), whereas all others were vaccinated with BNT162b2. Results of SARS-CoV-2 IgG antibodies in BAU/mL and the respective neutralizing capacity in % after the third boost vaccination are shown in categories in numbers and % in Table 1 and Figure 1. These data, measured 89 ± 32 days after the third boost vaccination, were compared to the respective data measured 96 ± 63 days after the second immunization. The results demonstrate a clinically relevant increase in proportions reaching presumed protective antibody titers above 216 BAU/mL (Chi-Square test *p* < 0.001) as well as an increase in neutralizing antibody titers from 68 ± 33% to 89 ± 24% (paired T-test, *p* < 0.001). 

### 3.2. Risk Factor Analysis for Immunological Non-Response (**B**) 

*Group A* This group consisted of 153 patients with routine standard third dose vaccination of which only 12 patients (7.8%) fulfilled the definition criteria for non-response (drop in neutralizing titer levels <50%). On univariate logistic regression analysis time from third boost vaccination to laboratory measurement of antibodies (RC-B = 0.023, *p* = 0.004, Exponent-B = 1.023, 95% CI 1.007–1.040), age (RC-B = 0.052, *p* = 0.041, Exponent-B = 1.053, 95% CI 1.002–1.160) and presence of malignancy (RC-B = 1.924, *p* = 0.038, Exponent-B = 6.850, 95% CI 1.116–42.055) were identified as potential risk factors for non-response and were incorporated in a multivariate regression model. Here, given the close 95% confidence interval and *p*-level, multivariate logistic regression analysis pointed towards time between last booster vaccinations to antibody measurement as an important risk factor (Table 2a). If univariate analysis was used as the basis for calculation, the probability (odds) of a drop in neutralizing titers below 50% after 100 days increased by a factor of 4.9. 

*Group B* This group consisted of 163 patients with routine standard third dose and/or need for repeat booster vaccinations ≥4 of which 22 patients (13.5%) fulfilled the definition criteria for non-response (drop in neutralizing titer levels <50% and/or need for repeat vaccination). On univariate logistic regression analysis, time from third/last boost vaccination to laboratory measurement of antibodies (RC-B = 0.014, *p* = 0.029, Exponent-B = 1.015, 95% CI 1.001–1.028), age (RC-B = 0.057, *p* = 0.004, Exponent-B = 1.058, 95% CI 1.018–1.100), presence of malignancy (RC-B = 2.030, *p* = 0.007, Exponent-B = 7.611, 95% CI 1.749–33.117), and presence of immunosuppressive therapy (RC-B = 1.646, *p* = 0.002, Exponent-B = 5.184, 95% CI 1.852–14.508) were identified as potential risk factors for non-response and were incorporated in a multivariate regression model. On multivariate regression analysis (Table 2b), age and presence of immunosuppressive medications remained strong predictors for an early drop in neutralizing antibody titers and/or vaccination failure even after three dosages.

### 3.3. Sensitivity Analysis

We performed a stepwise forward logistic regression analysis to confirm the results using the following variables: BMI, eGFR, albumin, time from last vaccination to laboratory measurement, age, dialysis vintage, sex, prior renal transplant, diabetes mellitus, presence of malignancy, and presence of immunosuppressive medication. Here, again only age (RC-B = 0.072, *p* = 0.001, Exponent-B = 1.074, 95% CI 1.028–1.122) and presence of immunosuppressive therapy (RC-B = 2.248, *p* ≤ 0.001, Exponent-B = 9.473, 95% CI 2.770–32.398) increased the probability of non-response to vaccination defined as a neutralizing antibody titer <50% and/or need for repeat vaccination. An increase in age of a decade doubled the probability (odds) of reaching the outcome parameter by 2.041.

### 3.4. Effect of ≥4 Booster Vaccinations on SARS-CoV-2 IgG Antibodies and Their Neutralizing Capacity in Low- and/or Non-Responders (**C**)

Second, we analyzed ten patients with failure to mount adequate SARS-CoV-2 antibody titers even after a third booster vaccination, who then received a fourth (9 pts) and even a fifth (1 pt) vaccination. Characteristics of these patients and of the course of the breakthrough infection are shown in Table 3. This case series differed from the patients without infections mainly in the following characteristics: presence of immunosuppressive therapy (60.0% vs. 10.5%), diabetes mellitus (20.0% vs. 30.1%), time on dialysis (3.2 vs. 5.5 years), and age (87.8 vs. 67.4 years), but did not differ with respect to time elapsed from last repeat booster vaccination to laboratory measurement (87 vs. 89 days). Surprisingly, except two patients, all other patients were able to form protective antibody titers after repeat vaccination using an mRNA-based vaccine for repeat booster immunization comparable to titers of the group of patients with only one booster vaccination (82% vs. 89%). Finally, we could not find any clinically negative effects or safety concerns of repeat vaccinations in these patients. 

### 3.5. Clinical Course of Breakthrough Infections in 20 Patients on Chronic Hemodialysis (**D**) 

Data on 20 patients (mean age 62 years, range 38–85) with PCR proven SARS-CoV-2 breakthrough infections and full recovery were available at the time of submission of this manuscript (Table 4). On diagnosis, mean CT-levels were 16 (range 9–21) and mean neutralizing antibody titer was 83% (range 0–100). The time from diagnosis to complete viral clearance on PCR testing, or PCR CT-levels >33, ranged from 7 to 29 (mean 13) days. Without exception, all patients had a clinically mild courses with no evidence of lower respiratory tract infection. Reported symptoms were flu-like sensations, runny noses, cough with or without sneezing, and mild headaches, or even no symptoms at all. Our case series consisted mainly of male participants (75%). We interpret this male preponderance as part of a selection bias unless further data, e.g., altered risk behavior as compared to women, should confirm male sex as a major risk factor for re- or breakthrough infections with the SARS-CoV-2 variants. At the time of submission, another seven patients are PCR-positive. Three are currently not specifically treated, but one patient suffering from multiple myeloma received i.v. antibodies against SARS-CoV-2. All patients are clinically asymptomatic or show only mild symptoms.

## 4. Discussion

The results of this investigation (**A**) demonstrate a high efficacy of third booster vaccinations in safely inducing neutralizing SARS-CoV-2 antibodies, (**B**) confirm age and the presence of immunosuppressive medication as significant risk factors for reduced vaccine response, (**C**) suggest a safe efficacy of fourth vaccinations as well as a reduction of immunosuppressive therapy as effective measures in persons with previous vaccination failure, and (**D**) present preliminary data on probably clinically mild disease progressions and prolonged viral shedding in hemodialysis patients infected with SARS-CoV-2 omicron variant despite prior boost vaccinations. 

Novel mRNA-based vaccines intriguingly resulted in higher seroconversion rates in hemodialysis patients as compared to effects of so far classically manufactured standard vaccines, e.g., against Hepatitis B. Accordingly, one study reported pooled estimates of response rates of 45% and 89% after the first and second dose, respectively [16]. First data on the effect of SARS-CoV-2 booster vaccination in dialysis patients were reported in July 2021 [17]. In this study, 88 chronic haemodialysis patients received booster vaccinations with BNT162b2 mRNA vaccine following a standardised vaccination protocol three months after the second dose. All but two participants showed a significant increase in SARS-CoV-2 specific antibody titers by a factor of approximately 71 from 55 to 3900 U/mL. The only two patients in this study who did not show an adequate increase in antibody titers had received active immunosuppressive therapy. In the meantime, the omicron variant has almost completely displaced other virus variants, at least in Germany [15]. In Germany, third dose booster vaccinations in hemodialysis patients were officially approved in October 2021. First reports on the effect of booster vaccination in dialysis patients date from early 2022. One of the first investigations examined 50 patients on average 158 days after booster vaccination and found median neutralizing titers of 282 [18]. Another clinical study evaluated the humoral response in 38 hemodialysis and 31 peritoneal dialysis patients 30 days after three doses of BNT162b2 [19]. The authors could report also on significant increases in antibody titers (data reported in AU/mL). Furthermore, twelve former low-responders and two of three non-responders (after two doses) showed significant increases in their antibody levels after the third booster vaccination. Since then, further investigations on the efficacy of booster vaccinations in end-stage renal disease patients have been published [20,21,22,23,24]. These investigations evaluated IgG antibody titers in cohorts of 40 to over 2700 hemodialysis patients. Comparable to our data, these analyses also showed a clear titer increase in over 90% of the participants. Our data are in line with these studies indicating titer levels after boost vaccination clearly above levels following the second standard dose and significant increases even in former non- or low-responders. Nevertheless, comparability between investigations is still hampered by the fact that antibody levels were measured at different time points after booster vaccinations, applied assays, and a lack of standardization of outcome parameters (e.g., definition of low-response or partial response etc.). Our results appear to essentially confirm these data but add to the findings an analysis of the SARS-CoV-2 omicron variant neutralizing capacity of the antibodies. However, comparison between these studies seems to be complicated by differences in assays and laboratory units. Another major difference in study design and a major limitation of our analysis is the fact that we could not titrate the antibody titers towards maximum values but could only determine them up to a threshold of 433 BAU/mL. Therefore, we cannot comment on titer evolution in patients with third booster vaccinations who already had titers higher than 433 BAU/mL after the second dose. Nevertheless, one might speculate that repeat vaccinations may result in decreased waning of antibody titers as described in different other patient populations [25] and dialysis patients [26,27]. Despite rapid titer waning, Clarke et al. were able to confirm a longstanding SARS-CoV-2 antigen–specific T-cell response in patients without detectable antibody titers six months post vaccination [28]. Therefore, antibody titer measurement alone is obviously not enough to assess a potential immune response to future infections with SARS-CoV-2 viruses. Nevertheless, our study demonstrates that only 10.5% of patients showed antibody levels below a threshold of 216 BAU/mL, a level roughly considered protective against a symptomatic infection [29]. Unfortunately, due to the large number of commercially available test assays with different cutoff and reference range values, comparisons between these studies are hampered. Therefore, the World Health Organization has made attempts [30] to standardize titer reporting in binding antibody units (BAUs). 

So far, many risk factors for non-response to vaccination, e.g., reduced serum albumin levels, immunosuppressive therapy, age, high dialysis vintage, and low IgG levels, have been proposed [31,32,33]. Our data confirm age and immunosuppression as important risk factors for non-response but not albumin or dialysis vintage. The reasons for this are unclear but may most likely be due to differences in study designs, laboratory assays, patient selection, low patient numbers, or statistical analysis methods. Nevertheless, older age could justify more intensive monitoring of antibody levels in this vulnerable patient population. 

Surprisingly, we were able to demonstrate the efficacy of a fourth vaccine administration in eight out of ten patients with low or non-response after two or three prior doses, respectively. One patient of our case-series was able to form effective antibody titers after transplant nephrectomy and subsequent slow withdrawal of tacrolimus and one complete non-responder with assumed immunosenescence was 88 years old. Nevertheless, another patient showed partial response to repeat vaccination despite an age of 96 years. In another patient, the dosing schedule of daratumumab was extended. Therefore, these preliminary data may cautiously suggest a repeat fourth vaccination in low- or non-responders on chronic hemodialysis. 

Our small case-series of breakthrough infections presents further preliminary data on SARS-CoV-2 infections in chronic hemodialysis patients. The clinically mild upper respiratory tract infections in this case-series give reason for hope for benign clinical courses also in the vulnerable population of chronic hemodialysis patients and are in line with most recent large population-based investigations. Nevertheless, some of the patients did show clinical signs of systemic infection as low-grade fever, headaches, or reported a general feeling ill without further specification, whereas others were remarkably asymptomatic. Of note, one patient died of other causes while tested positive with SARS-CoV-2 while another patient with myeloma experienced unremarkable viral clearance after treatment with i.v. SARS-CoV-2 antibodies. This patient showed normal results on chest X-ray and pulmonary CT excluding severe lower respiratory tract involvement. These data must be interpreted in line with data showing a reduced antibody formation against the omicron variant after mRNA- and vector-based vaccinations (immune-escape). In the general population, the neutralizing capacity against omicron after three mRNA-based vaccinations is about the same as that against the delta variant after two vaccinations [34]. Recently, it could be shown in a nested-case control analysis in 56 dialysis patients with breakthrough infections that antibody levels in these individuals were significantly lower than in the unaffected control group [35], but these cases were documented before 14 September 2021, assuming that infection with omicron variants was unlikely.

Therefore, further studies on current disease and antibody patterns in hemodialysis patients are urgently needed to allow a better assessment of the future risk in this patient population as well as to adapt current hygiene measures in dialysis centers. For example, a study in over 2.2 million people in Qatar demonstrated a significantly milder clinical course of SARS-CoV-2 infection in individuals with booster vaccinations than in individuals with only two standard doses [36]. Comparable data have been reported from a large database of ~700,000 participants from Singapore [37]. Furthermore, this investigation showed higher antibody titers in patients after heterologous mRNA-based vaccination schemes as compared to homologous mRNA vaccinations. Another epidemiological study provided evidence for the effectiveness of booster vaccinations against severe SARS-CoV-2 B.1.1.529 (Omicron) infections [38]. To counteract the immune escape of the B.1.1.529 variant against previous mRNA-based vaccines, announcements of a vaccine adaptation have already been published [39], but so far, no definite results have been available. Now a new and even more infectious BA.2 variant is the most prevalent strain in Germany and worldwide. Our initial data on mild courses of infection in dialysis patients support most recent publications on 23 hemodialysis patients without any need for hospitalization [40] and give hope that the Omicron wave will not be associated with high hospitalization and mortality rates in this highly vulnerable patient group.

In summary, booster vaccinations with current mRNA-based vaccines provide reasonable protection against severe SARS-CoV-2 B.1.1.529 (Omicron) infections also in chronic hemodialysis patients. Age and immunosuppressive drugs remain relevant risk factors for vaccination non-response not only after standard two dose vaccination but also after repeat administration. Breakthrough infections with B.1.1.529 will probably have a clinically mild course in most cases. 

### Major Limitations

Interpretation of the results of this investigation is limited by several factors: (i) observational study design with relatively low patient numbers; (ii) only a few participants experienced the predefined outcome parameters; (iii) due to the local and geographic restriction of the study design, applicability of the results in international settings is impaired; (iv) we were not able to measure T-cell mediated immune responses; (v) antibody titers were collected during routine patient care and could not be titrated manually, so a cut-off value of >433 BAU/mL was introduced; (vi) given the high drop-out rates and the voluntary participation, potential for final selection bias of study participants could not be ruled out definitely; (vii) we were not able to compare the data in hemodialysis patients with a control group; (viii) there was variability in time between vaccine administration and serology; and (ix) infections were proved on viral PCR-testing without further subtype determination. Nevertheless, subtype reporting by the central laboratory had only been abandoned after Omicron variants acceded >90% of subtypes.

## 5. Conclusions

This study demonstrates high success rates of neutralizing antibody formation in chronic dialysis patients after application of a standard third mRNA-based booster vaccination. Advanced age and therapy with immunosuppressive drugs were major risk factors for vaccination failure. However, in selected cases, an additional fourth dose may ensure a safe titer increase in neutralizing antibodies without noticeable side effects. Therefore, repeated vaccination and reduction in immunosuppressive medications, if possible, are options to overcome vaccination failure in selected patients. Furthermore, these preliminary outcome data on breakthrough infections with SARS-CoV-2 Omicron in hemodialysis patients seem to give hope for an overall clinically mild infection also in this vulnerable patient population. Future data on the spread of Omicron among hemodialysis patients, staff members, and outcome are needed to adapt hygiene concepts and aid in patient counselling. 

## Figures and Tables

**Table 1:** Effect of second and third (booster) vaccinations on SARS-CoV-2 IgG antibody levels and neutralizing capacity in categories in 153 patients on chronic hemodialysis.

	Categories	Post Second Vaccination (No, %)		Post Third Vaccination (No, %)		Somers‘d
**IgG**	>433	84	54.9%	127	83.0%	<0.001
	216–433	10	6.5%	10	6.5%	
	<216	59	38.6%	16	10.5%	
**% inhibition**	>66	99	64.7%	136	88.9%	0.003
	33–66	23	15.0%	7	4.6%	
	<33	31	20.3%	10	6.5%	

Data are shown in numbers and % after the second and third vaccination. The variables were categorized into three parts each. SARS-CoV-2 IgG antibody titers are shown in BAU/mL, whereas the neutralizing capacity of the IgG antibodies is shown in % inhibition. Mean time between the two laboratory measurements was 158 ± 59 days. Somers’s test was applied to compare ordinal categories before and after the boost vaccination. A *p*-level of <0.05 was considered statistically significant. A paired t-test was applied to compare the neutralizing capacity of SARS-CoV-2 IgG antibodies (%) before (68 ± 33) and after (89 ± 24) the boost vaccination (*p* < 0.001).

**Table 2:** Multivariable logistic regression analysis of risk factors for vaccination non-response in 153 HD-patients (group A) after three (a) or in 163 HD-patients (group B) after three and more (b) SARS-CoV-2 vaccinations.

**(a)**					
**Variable**	**RC-B**	***p*** **-Level**	**Exponent-B**	**95%-CI**	
boost to lab (days)	0.016	0.082	1.016	0.998	1.034
Age (years)	0.034	0.200	1.035	0.982	1.090
Active malignancy	1.142	0.300	3.134	0.362	27.170
**(b)**					
**Variable**	**RC-B**	***p*** **-Level**	**Exponent-B**	**95%-CI**	
boost to lab (days)	0.008	0.287	1.008	0.993	1.023
Age (years)	0.064	0.004	1.066	1.020	1.114
Active malignancy	0.458	0.617	1.581	0.263	9.494
isMedication	2.112	0.002	8.267	2.206	30.975

(a) Variables with evidence of a relevant influence on the outcome parameter on univariable regression analysis were then examined in a multivariate model. Here, only time elapsed from third booster vaccination to laboratory measurement of SARS-CoV-2 antibody titers showed a meaningful impact on the outcome parameter defined as neutralizing antibody titers <50%. RC-B = regression coefficient, 95%-CI = 95% confidence interval of exponent-B. (b) Variables with evidence of a relevant influence on the outcome parameter on univariable regression analysis were then examined in a multivariate model. Here, only the factors age and presence of immunosuppressive medication were identified as risk factors for vaccination non-response defined as “neutralizing antibody titers below 50% and/or need for repeat vaccination” even after a third booster vaccination. RC-B = regression coefficient, 95%-CI = 95% confidence interval of exponent-B. Data of a sensitivity analysis are presented in the results section.

**Table 3:** Characteristics of ten chronic hemodialysis patients with 4–5 repeat vaccinations after non-response to 3 standard SARS-CoV-2 vaccine doses.

No.	Age	BMI	Diabetes	pKTx	Vintage	Vaccination	isMeds	aTumor	Albumin	Kt/V	IgG	nTiter
1.	50–55	23.0	/	Yes	1.59	5 times mRNA	ldST/Tacrolimus	/	2843	1.1	>433	87
2.	85–90	32.4	/	/	0.93	4 times mRNA	ldST	Yes	3116	1.1	0	5
3.	65–70	27.7	/	/	3.70	4 times mRNA	/	/	3624	1.4	>433	94
4.	95–100	30.6	/	/	1.77	1 vector + 3 mRNA	ldST	/	3526	0.8	211	88
5.	60–65	31.3	/	/	1.11	4 times mRNA	ST/Cyclophosphamide	/	3481	1.1	>433	98
6.	85–90	25.7	/	/	2.38	4 times mRNA	/	/	3829	0.9	>433	100
7.	80–85	23.7	Yes	/	5.82	4 times mRNA	/	/	3069	1.0	>433	100
8.	75–80	23.8	/	/	6.12	4 times mRNA	ST/Daratumumab	Yes	3450	1.3	>433	99
9.	80–85	20.0	Yes	/	5.91	4 times mRNA	/	/	3330	1.4	>433	58
10.	90–95	22.2	/	/	2.69	4 times mRNA	ldST	/	3333	0.9	>433	83

Age in years, BMI = body mass index in kg/m^2^, pKTx = prior kidney transplant, vintage = time on dialysis in years, isMeds = presence of immunosuppressive medication, ST = steroids, ldST = low-dose steroids, aTumor = active malignancy, IgG = SARS-CoV-2 IgG antibody titers in BAU/mL, nTiter = neutralizing titer (capacity) of IgG antibodies in %. Laboratory evaluation was performed an average 87 ± 40 days after the last vaccination. Patient number 1. responded to repeat vaccination after transplant nephrectomy and discontinuation of tacrolimus, patient number 2. suffered from smoldering prostate cancer, and patient number 4. did not mount adequate antibody levels after repeat vaccination presumably due to immunosenescence and longtime steroid medication, whereas all other patients were able to mount protective levels of SARS-CoV-2 antibodies after four vaccinations. Of note, even patients on prior immunosuppressive medication mounted protective antibody titers after cessation of the respective drugs (tacrolimus was stopped after transplant nephrectomy, cyclophosphamide was stopped after renal failure due to systemic vasculitis, and daratumumab was reduced by increasing application intervals). Multiple vaccine administrations also resulted in no measurable side effects or safety concerns, 60% of pts were male.

**Table 4:** Clinical characteristics and course of SARS-CoV-2 breakthrough infections in 20 patients on chronic hemodialysis.

No.	Age	BMI	Diabetes	pKTx	Vintage	Vaccination	isMeds	IgG	nTiter	CT	Remission/Days	Course
1.	50–55	22	No	Yes	7.2	3 times mRNA	ldST	>433	100	21	18	Mild
2.	55–60	31	No	Yes	10.5	3 times mRNA	no	>433	100	17	14	Mild
3.	55–60	29	No	No	2.1	3 times mRNA	no	0	<30	17	16	Mild
4.	30–35	19	No	Yes	5.8	2 times mRNA	no	>433	98	22	7	Mild
5.	60–65	35	Yes	No	0.4	2 times mRNA	no	70	48	9	29	Mild
6.	70–75	28	Yes	No	1.6	wild, 2 times mRNA	no	>433	84	15	12	Mild
7.	50–55	32	Yes	No	4.0	3 times mRNA	no	>433	99	13	8	Mild
8.	35–40	31	Yes	Yes	2.1	3 times mRNA	no	>433	100	18	13	Mild
9.	80–85	19	No	No	3.4	3 times mRNA	no	>433	100	18	13	Mild
10.	30–35	20	Yes	No	0.7	3 times mRNA	no	>433	97	14	15	Mild
11.	80–85	24	No	No	0.9	4 times mRNA	no	>433	99	16	14	Mild
12.	70–75	24	No	No	4.4	3 times mRNA	no	>433	100	16	12	Mild
13.	75–80	20	No	No	7.9	3 times mRNA	no	>433	99	13	11	Mild
14.	60–65	30	Yes	No	0.8	3 times mRNA	ldST	372	51	19	8	Mild
15.	60–65	25	No	No	3.8	3 times mRNA	No	>433	98	21	7	Mild
16.	50–55	27	No	Yes	1.6	4 times mRNA	No	>433	97	18	10	Mild
17.	70–75	23	No	No	14.7	3 times mRNA	No	>433	99	16	12	Mild
18.	60–65	32	No	No	1.1	3 times mRNA	ldST	>433	98	19	12	Mild
19.	75–80	25	No	No	8.7	3 times mRNA	No	>433	100	12	8	Mild
20.	80–85	19	Yes	No	0.6	1 time mRNA	ST	0	0	14	12	Mild

Age in years, BMI = body mass index in kg/m^2^, pKTx = prior kidney transplant, vintage = time on dialysis in years, isMeds = presence of immunosuppressive medication, wild = infection before vaccination, ldST = low-dose steroids ≤5 mg, IgG = SARS-CoV-2 IgG antibody titers in BAU/mL, nTiter = neutralizing titer (capacity) of IgG antibodies in %, CT = crossing threshold-level on PCR-testing at time of diagnosis, remission = time from first diagnosis to complete PCR proven resolution in days (weekly measurements), one patient was accidentally diagnosed on hospital admission, all others on routine pre-dialysis testing. Mean time to complete remission of PCR testing was 14 days. No patient developed signs of lower respiratory tract infection by SARS-CoV-2 or was hospitalized due to SARS-CoV-2 infection of the upper airways. One patient died of other causes while tested positive with SARS-CoV-2. This patient showed normal results on chest X-ray and pulmonary CT. One patient with myeloma received SARS-CoV-2 specific i.v. antibodies, 75% of pts were male.

## Data Availability

The data underlying this article cannot be shared publicly for the privacy of some individuals that participated in the study. The data will be shared on reasonable request to the corresponding author. The results presented in this paper have not been published previously in whole or part, except in abstract format.
